# Tumor size and stage assessment accuracy of MRI and ultrasound versus pathological measurements in early breast cancer patients

**DOI:** 10.1186/s12905-025-03679-2

**Published:** 2025-04-04

**Authors:** Yuanyuan Liu, Xuerui Liao, Yakun He, Fawei He, Jing Ren, Peng Zhou, Xin Zhang

**Affiliations:** 1 Department of Radiology, Sichuan Clinical Research Center for Cancer, Sichuan Cancer Hospital & Institute, Sichuan Cancer Center, University of Electronic Science and Technology of China, 55 # South Renmin Road, Chengdu, 610041 Sichuan China; 2 Department of Breast Surgery, Sichuan Clinical Research Center for Cancer, Sichuan Cancer Hospital & Institute, Sichuan Cancer Center, University of Electronic Science and Technology of China, 55 # South Renmin Road, Chengdu, 610041 Sichuan China; 3 Department of Ultrasound, Sichuan Clinical Research Center for Cancer, Sichuan Cancer Hospital & Institute, Sichuan Cancer Center, University of Electronic Science and Technology of China, Chengdu, 610041 China

**Keywords:** Breast cancer, Tumor size, Tumor stage, Magnetic resonance imaing, Ultrasound

## Abstract

**Background:**

Accurate size and stage estimation is important to monitor tumor response and plan further treatment in breast cancer patients undergoing neoadjuvant chemotherapy. To evaluate the accuracy of imaging findings [ultrasound (US) and magnetic resonance imaging (MRI)] for tumor size and stage estimations in early breast cancer patients and to elucidate the factors influencing tumor stage assessment.

**Methods:**

We retrospectively enrolled consecutive women having pathologically confirmed breast cancer (stage T1/T2, 199 patients and 201 lesions) and preoperative records available for both US and MRI. The concordance between imaging-determined and pathological tumor size and stage was explored. The McNemar’s test was conducted to compare the concordance between imaging-determined tumor size and imaging-determined tumor stage. Multivariate logistic regression was used to analyze the factors that influenced the accuracy.

**Results:**

The concordance between US-determined and pathological tumor size (71.1%) was comparable to MRI–pathology concordance (72.6%). MRI–determined stage concordance (73.6%) was comparable to US-determined stage concordance (69.2%). Tumors with a larger pathological size, were more likely to be underestimated by US or MRI in terms of tumor size and stage (all *P* < 0.05).

**Conclusion:**

Tumor size and tumor stage concordance did not significantly differ between US and MRI in early breast cancer patients; US could be the first choice for tumor size estimation and tumor staging.

## Background

Breast cancer is the most common cancer among women worldwide [1]. Accurate preoperative estimation of the tumor size is instrumental in tumor stage estimation, planning the treatment, and ensuring negative resection margins [2, 3]. In particular, in patients scheduled to undergo neoadjuvant chemotherapy, accurate size and stage estimation is important to monitor tumor response and plan further treatment [4, 5]. Owing to its convenience and cost-effectiveness, ultrasound (US) is the most commonly used imaging modality for tumor size estimation, particularly in developing countries like China. Most studies found that US underestimated the tumor size when compared with pathological tumor size [6–9]. However, Xu et al. report that this underestimation of tumor size has now reduced, probably because of upgraded equipment and better experience of doctors performing US [8]. MRI is reportedly more accurate than US for tumor size estimation and is more sensitive to multifocal or multicentric lesions; MRI is also more likely to overestimate the tumor size [9–11]. However, some studies have reported contradictory results– US was better than MRI for tumor size estimation [12, 13].

When making comparative interpretations, some aspects may influence tumor size estimation, such as differences in the study designs and differences among participants [e.g., different breast histopathological types: invasive carcinoma, ductal carcinoma in situ (DCIS) or invasive lobular carcinoma (ILC)]. Limits of deviation within which US and MRI measurements are considered concordant with pathological tumor size measurements varied from 0 mm to 20 mm [10–21]. Furthermore, different MRI sequences and different measurements may influence the estimated tumor size [14, 19, 21]. Taken together, these abovementioned differences in studies may lead to different results, making these studies and their findings incomparable. Furthermore, Sogunro et al. [22] showed that no one single modality is the most accurate for detecting tumor size. However, tumor size helps determine the tumor stage, and clinical tumor stage is an important criterion for decision-making in clinical practice. For example, in T1 or T2 breast cancer patients, if there are two or fewer metastatic sentinel lymph nodes (SLNs), axillary lymph node dissection (ALND) may be avoided; in T3 patients, neoadjuvant chemotherapy could be the first choice. To our knowledge, most previous studies have focused on tumor size and few studies have explored the accuracy of imaging-determined tumor stage compared with pathological findings and compared the tumor stage estimation accuracy between US and MRI. As previous studies [8–11, 20] have shown that when tumor size was larger, the discordance of imaging-determined tumor size with pathology was higher, we focused on the tumor stage estimation in early breast cancers.

This study had the following purposes: (1) to calculate the concordance rates of US- and MRI-determined tumor sizes with pathological tumor size as well as the concordance rate of US- and MRI-determined tumor stage with pathological tumor stage; (2) to compare the accuracy of tumor size and stage estimation between US and MRI; and (3) to explore factors that influence the concordance between pathological and imaging-determined tumor size measurements and stage estimation.

## Methods

### Subjects

This retrospective study was approved by the Institutional Review Board of Sichuan Cancer Hospital & Institute (No. SCCHEC2015029). We reviewed our database and selected consecutive women with pathologically confirmed breast cancer between November 2017 and December 2019. The inclusion criteria were as follows: (1) pathologically confirmed breast cancer with stage T1 or T2; (2) use of both dynamic contrast-enhanced (DCE)-MRI and US as diagnostic methods before surgery; (3) undergoing surgical treatment with the availability of complete data of pathology (including tumor size). We excluded patients having (1) undergone biopsy before DCE-MRI and US examination; (2) undergone neoadjuvant chemotherapy or radiotherapy before surgery; (3) DCIS; and (4) reports with poor imaging quality or incomplete clinical data. Patients having undergone biopsy before DCE-MRI and US examination were excluded because the inflammation around the lesion caused by performing the biopsy would have led to inaccurate measurements and overestimation of the lesion size, thus obfuscating the findings. Our study was aimed at estimating the accuracy of imaging-determined tumor stage, and since DCIS is not classified in any tumor stage, we excluded it. Finally, 199 female breast cancer patients and 201 lesions were included.

Relevant demographic and clinical information was collected, including age, histopathological features, and tumor size on MRI, US, and pathology.

### Ultrasound

**Fig. 1:**
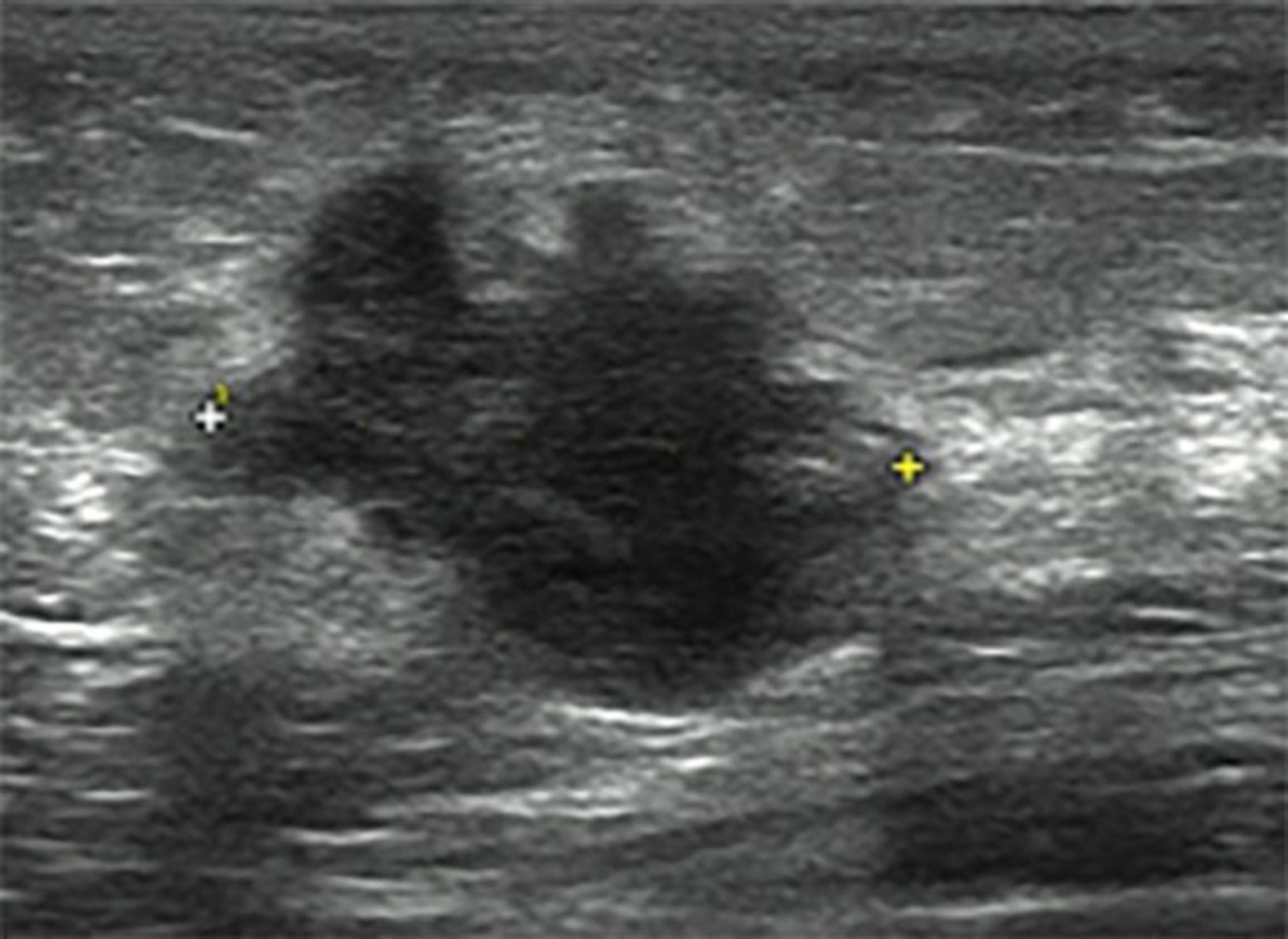
US image of a 54-year-old woman with no special type invasive breast cancer. The tumor size was 20 mm * 15 mm * 23 mm, and the largest diameter noted in any plane was considered to be the US-determined tumor size (23 mm)

All US examinations were conducted by dedicated US breast imaging doctor. A LOGIQ 9 (General Electric Medical System, Milwaukee, WI, USA) or EPIQ 7 (Philips Medical System, Bothell, WA, USA) scanner equipped with a 5–12-MHz linear array transducer was used for real-time scanning. One US doctor with 8 years of breast US experience retrieved images and remeasured the tumor size. Three measurements of the tumor were obtained in transverse, anteroposterior, and sagittal planes. The largest diameter noted in any plane was considered the US-determined tumor size (Fig. 1). Then we calculated the concordance rate of US-determined tumor sizes with pathological tumor size as well as the concordance rate of US-determined tumor stage with pathological tumor stage.

### MRI acquisition

All patients underwent MRI scanning with a 3.0-T Skyra device (Siemens Healthcare, Erlangen, Germany) with a dedicated 16-channel breast array coil; prone position was used for all MRI examinations. All MRI acquisition parameters are shown in Table 1.

**Table 1:** MRI protocol used in the study

MRI protocol	TR (ms)	TE (ms)	FOV (mm)	Slice Thickness (mm)	Matrix	Total Acquisition Time
Pre-contrast-enhanced sequences						
Axial T2WI	3570	70	340 × 340	4	358 × 448	3 min 29 s
Sagittal T2WI	3600	56	200 × 200	3	358 × 448	2 min 47 s
T1 mapping	5.64	2.46/3.69	360 × 360	2.5	269 × 384	58 s
**Post-contrast-enhanced sequence**						
CAIPIRINHA-Dixon-Twist-Vibe	5.64	2.46	360 × 360	2.5	269 × 384	5 min 12 s
**Delayed contrast-enhanced sequence**	8.73	4.41	340 × 340	0.8	408 × 448	3 min 45 s

TR, repetition time; TE, echo time; FOV, field of view; T2WI, T2-weighted imaging

### MRI analysis

**Fig. 2:**
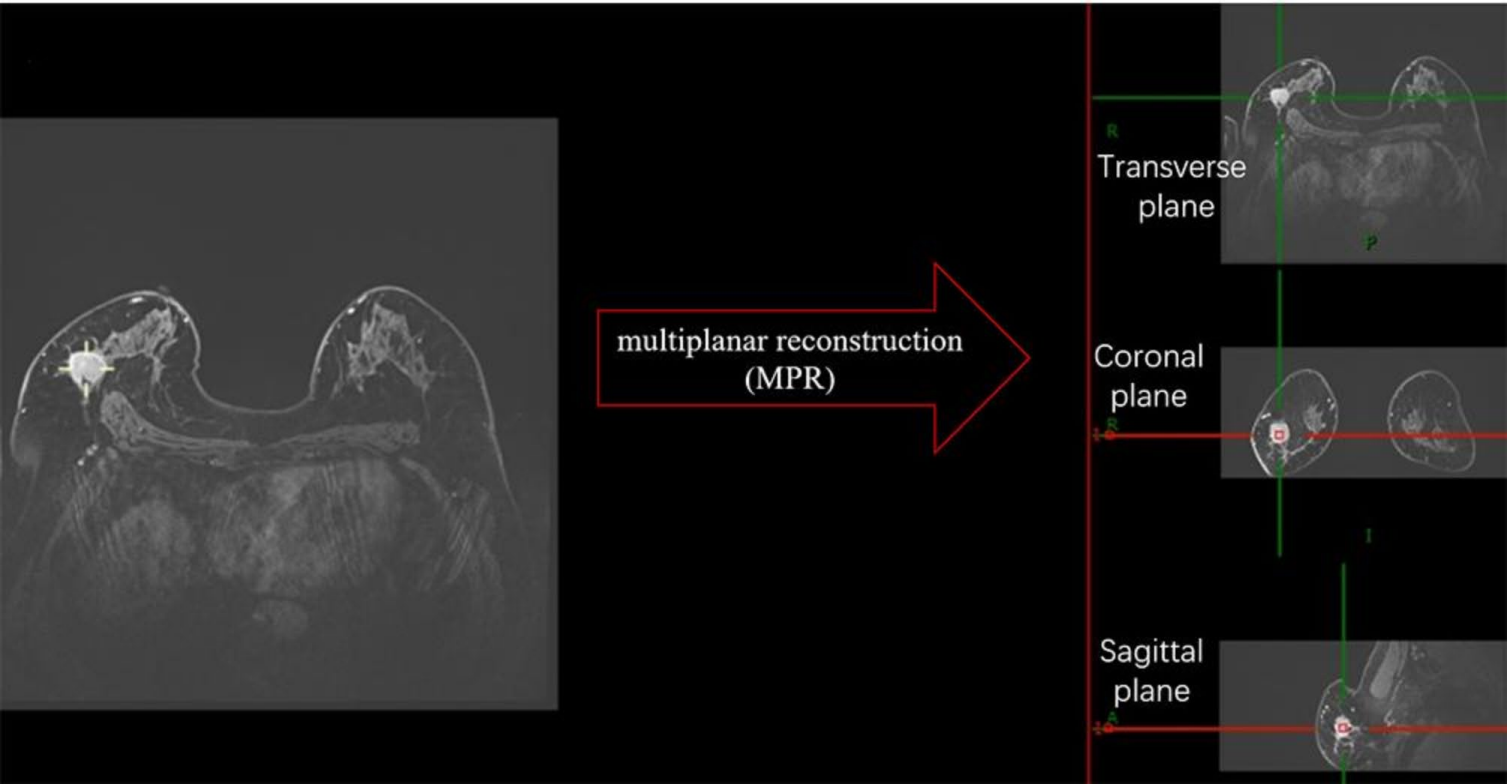
MR images of the same 54-year-old woman with no special type invasive breast cancer: breast tumor size measurements with MRI through multiplanar reconstruction (MPR)

**Fig. 3:**
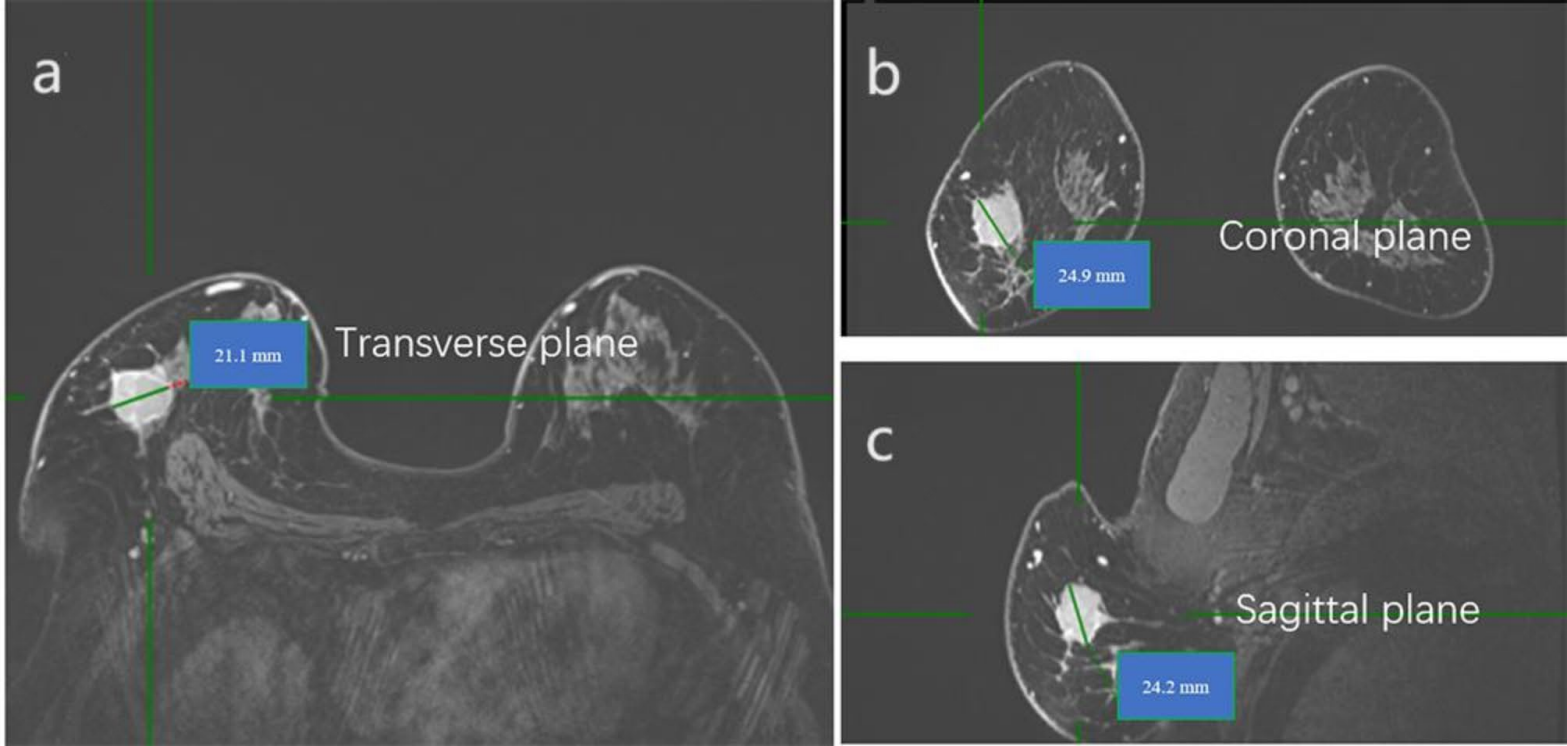
MR images of the same 54-year-old woman with no special type invasive breast cancer. **(a)** the largest diameter in the transverse plane (21.1 mm), representing MRI-Trans; **(b)** the largest diameter in the coronal plane (24.9 mm); and **(c)** the largest diameter in the sagittal plane (24.2 mm). The diameter in the coronal plane (24.9 mm) is the largest; thus, the diameter of MRI-L is 24.9 mm

Two radiologists with 10 and 8 years of experience in analyzing breast MRI reviewed the images and then measured tumor size. In the delayed phase of dynamic studies, maximal tumor extension was measured in the anatomical planes [transverse plane, coronal plane through multiplanar reconstruction (MPR), and sagittal plane through MPR, Fig. 2]. Because the slice thickness in the delayed phase was 0.8 mm, it allowed for a more detailed measurement of the tumor and MPR. When we measured tumor size in the delayed phase, we used tumor size in the peak enhancement phase as a reference standard to exclude surrounding blood vessels and enhancement background, which may lead to tumor size overestimation. MRI-Trans was defined as the maximal diameter in transverse plane. MRI-L was defined as the largest diameter in any plane (Fig. 3). Next, we calculated the concordance rate of MRI-determined tumor sizes (MRI-L and MRI-Trans) with pathological tumor size as well as the concordance rate of MRI-determined tumor stage with pathological tumor stage.

### Pathology

Tumors were sliced along their long axis, and the largest diameter was considered as the pathological tumor size and the gold standard measure of that tumor. Tumors were classified into four histopathological subgroups: invasive carcinoma of no special type (NST), invasive carcinoma with DCIS, ILC, and other types. Immunohistochemical staining was performed to identify the status of estrogen receptor (ER), progesterone receptor (PR), human epidermal growth factor receptor 2 (HER2), and Ki-67. Tumors were considered ER- or PR-positive if ≥ 10% of cells appeared immunostained. Similarly, Ki-67 positivity was considered as ≥ 14% cells appearing immunostained. HER2 positivity was defined as hematoxylin–eosin (HE) staining 3 + or HE staining 2 + with positive fluorescence in situ hybridization test.

### Statistical analysis

All statistical analyses were performed with SPSS (v19.0; Chicago, IL). Continuous variables were expressed as mean with standard deviation (SD) if the distribution was normal, and categorical data were expressed as numbers and percentages. Then, the lesions concordance rates by MRI or US were counted. The one-way analysis of variance test was used to detect differences among US-determined, MRI-determined, and pathological tumor sizes with an assumption of normal distribution. The McNemar’s test was used to analyze the differences in categorical data among groups. Finally, multivariate logistic regression analysis with clinical and pathological features was done for accurate evaluation of tumor stage on MRI or US. *P* < 0.05 was considered to indicate a significant difference.

## Results

The sample comprised 199 female breast cancer patients and 201 lesions; one patient had three lesions, each with a different histopathology type. The mean age was 50.6 ± 10.4 years. The histopathological types were invasive carcinoma of NST (*n* = 143), NST with DCIS (*n* = 38), ILC (*n* = 7), and other pathologies [mucinous carcinoma (*n* = 5), tubular carcinoma (*n* = 1), and papillary carcinoma (*n* = 7)]. Other clinicopathological features shown in Table 2.

**Table 2:** Clinical and pathological characteristics

	Mean ± SD (or *n*, %)
Age (years)	50.6 ± 10.4
Tumor size by US (mm)	20.5 ± 7.4
Tumor size by MRI-L (mm)	22.4 ± 7.3 ^a^
Tumor size by MRI-Trans (mm)	20.3 ± 7.2
Pathological tumor size (mm)	20.3 ± 7.0
Pathological tumor stage	
T1	129 (64.2)
T2	72 (35.8)
Histology type	
NST	143 (71.1)
NST with DCIS	38 (18.9)
ILC	7 (3.5)
Other types	13 (6.5)
ER^b^	
+	147 (73.1)
-	54 (26.9)
PR^b^	
+	139 (69.2)
-	62 (30.8)
HER-2^c^	
+	48 (23.9)
-	151 (75.1)
Ki-67 ^d^	
+	157 (78.1)
-	42 (20.9)
Molecular subtype	
Lumina A	105 (52.2)
Lumina B	42 (20.9)
HER2 +	21 (10.5)
TNBC	33 (16.4)
Axillary lymph node metastasis	
+	76 (37.8)
-	125 (62.2)

NST, invasive carcinoma of no special type; DCIS, ductal carcinoma in situ; ILC, invasive lobular carcinoma; ER, estrogen receptor; PR, progesterone receptor; TNBC, triple-negative breast cancer

^a^ MRI-L compared with pathological tumor size, *P* < 0.05

### Concordance rate between US and pathological tumor size and stage

The mean tumor size determined by US was comparable to the mean pathological tumor size (Table 2). The concordance rate between US-determined tumor size and the pathological tumor size was 7.5%. When concordance between US and pathology results was defined as a difference of ≤ 5 mm in tumor size, the concordance rate was 71.1% (Table 3). The concordance rate between the US-determined tumor stage and pathological tumor stage was 69.2%, and underestimation and overestimation rates were 11.4% and 19.4%, respectively.

**Table 3:** Concordance rates of US or MRI–determined tumor size with pathological tumor size and concordance rates of US or MRI–determined tumor stage with pathological tumor stage

	Underestimation *n* (%)	Concordance *n* (%)	Overestimation *n* (%)
US–Pathological tumor size	90 (44.8)	15 (7.5)	96 (47.8)
US–Pathological tumor size ± 5 mm	27 (13.4)	143 (71.1)	31 (15.4)
US–determined tumor stage	23 (11.4)	139 (69.2)	39 (19.4)
MRI-Trans–Pathological tumor size	91 (45.3)	21 (10.4)	89 (44.3)
MRI-Trans–Pathological tumor size ± 5 mm	25 (12.4)	146 (72.6)	30 (14.9)
MRI-Trans–determined tumor stage	17 (8.5)	148 (73.6)	36 (17.9)
MRI-L–Pathological tumor size	49 (24.4)	26 (12.9)	126 (62.7)
MRI-L–Pathological tumor size ± 5 mm	14 (7.0)	145 (72.1)	42 (20.9)
MRI-L–determined tumor stage	10 (5.0)	139 (69.2)	52 (25.9)

### Concordance rate between MRI and pathological tumor size and stage

The mean tumor size determined by MRI-L was significantly larger than the mean pathological tumor size, and the mean tumor size by MRI-Trans was comparable to the mean pathological tumor size (Table 2). The concordance rate was 10.4% between MRI-Trans-determined tumor size and the pathological tumor size and 12.9% between MRI-L-determined tumor size and pathological tumor size (Table 3). When concordance between MRI and pathology was defined as a difference of ≤ 5 mm in tumor size, the concordance rate, underestimation rate, and overestimation rate of MRI-Trans were 72.6%, 12.4%, and 14.9%, respectively. MRI-L showed a similar concordance rate of 72.1% but a higher overestimation rate (20.9%).

The concordance rate between MRI-Trans–determined tumor stage and pathological tumor stage was 73.6%, and underestimation and overestimation rates were 8.5% and 17.9%, respectively (Table 3). The concordance rate between MRI-L–determined tumor stage and pathological tumor stage was 69.2%, with a higher overestimation rate (25.9%) and a lower underestimation rate (5.0%).

### Concordance rate between MRI and US

There was no significant difference in tumor size concordance between US–pathology ± 5 mm and MRI-Trans–pathology ± 5 mm groups (*P* = 0.418), while the overestimation rate of MRI-L–pathology ± 5 mm group was higher than those of the other two groups (*P* < 0.001) (not shown in table).

There was no significant difference in tumor stage concordance between US and MRI-Trans, whereas significant differences were found between US and MRI-L and between MRI-Trans and MRI-L (Table 4), with MRI-L showing a higher overestimation rate than US and MRI-Trans.

**Table 4:** Concordance rate of tumor stage between MRI and US

		**MRI-Trans–determined tumor stage**			*P*	**Kappa**
		Underestimation	Concordance	Overestimation	0.275	0.521
US–determined tumor stage	Underestimation	12	11	0		
	Concordance	5	122	12		
	Overestimation	0	15	24		
		**MRI-L–determined tumor stage**			*P*	**Kappa**
		Underestimation	Concordance	Overestimation	< 0.001	0.445
US–determined tumor stage	Underestimation	8	15	0		
	Concordance	2	113	24		
	Overestimation	0	11	28		
		**MRI-L–determined tumor stage**			*P*	**Kappa**
		Underestimation	Concordance	Overestimation	< 0.001	0.717
MRI-Trans–determined tumor stage	Underestimation	10	7	0		
	Concordance	0	131	17		
	Overestimation	0	1	35		

### Factors influencing concordance rate

When concordance between US and pathology results was defined as a difference of ≤ 5 mm in tumor size, only pathological size had a significant impact on US-determined tumor size underestimation by multivariate regression analysis (Table 5); pathological size was also associated with US-determined tumor stage underestimation.

**Table 5:** Factors influencing concordance rate

		Influence factor	OR (95% CI)	*P*
US–Pathological tumor size ± 5 mm	Underestimation	Pathological size	8.041 (3.280–19.709)	< 0.001
US–determined tumor stage	Underestimation	Pathological size	4.135 (1.822–9.388)	0.001
MRI-Trans–pathological tumor size ± 5 mm	Underestimation	Pathological size	12.410 (4.464–34.505)	< 0.001
MRI-Trans–determined tumor stage	Underestimation	Pathological size	5.711 (1.974–16.524)	0.001
MRI-Trans–determined tumor stage	Overestimation	Negative PR	0.102 (0.019–0.540)	0.007

When concordance between MRI and pathology results was defined as a difference of ≤ 5 mm in tumor size, only pathological size had a significant impact on MRI-determined tumor size underestimation. Pathological size was significantly associated with MRI-Trans tumor stage underestimation; conversely, PR negativity was significantly associated with MRI-Trans tumor stage overestimation (Table 5).

All other factors, including age, histopathological type, ER status, HER2 status, Ki-67 levels, molecular subtype, and axillary lymph node metastasis, were not significantly associated with tumor size and stage concordance rate (not shown in table).

## Discussion

Our results showed that the concordance rate of US with pathological tumor size with a cutoff of 5 mm was 71.1%, which was in line with some of the previous reports [7, 10] but higher than some others [8, 11, 23, 24]. When the concordance between MR and pathology was defined as a difference of ≤ 5 mm in tumor size, the concordance rate was in agreement with some previous studies wherein it varied from 68.3 to 76.7% [18, 19, 25] but higher than some others (30.2–62.5%) [17, 19, 24]. The concordance rates of US–determined and MRI-Trans–determined tumor stage were 69.2% and 73.6%, and the accuracy in about 70% of cases was acceptable considering tumor shrinkage during fixation [26]. MRI-L–determined tumor size and stage both showed similar concordance rates but higher overestimation rates than US and MRI-Trans. Previous studies have reported that MRI is more likely to overestimate the tumor size; this could be because size measurements calculated using the largest diameter were used to reflect the size of the tumor in these studies [10, 14, 27]. The largest diameter on MRI may not offer the most accurate tumor size measurement and may consequently lead to tumor stage overestimation.

When MRI-Trans and US–determined sizes and stages were compared, there was no significant difference in the concordance rate of tumor size and stage between US and MRI-Trans. This result was different from previous studies. Some previous studies reported US being more accurate than MRI for predicting tumor size [12, 13], whereas others reported MRI being more accurate than US [9–11]. This disparity between our findings and previous reports may be due to our patient sample (early breast cancer, stage T1 or T2), our different size measurement on MRI (using MR-Trans and not MRI-L as the tumor diameter to avoid overestimation), and widespread use of US in China and extensive experience of our doctors in performing US, thus reducing underestimation. We suggested that US could be the first choice for tumor size estimation and tumor staging in early breast cancer patients, and it could act as an alternative to some MRI examinations. In addition, US also can be used to assess size between treatments and can thus help confirm downsizing shortly after the treatments, which is clinically relevant to switch chemo-lines beforehand. US can also serve as an essential complement to mammography during the screening phase, being both accurate and radiation-free. Taken together, US can save both time and healthcare costs, and it suggests that we can strongly advocate its use in regions with varying resources and expertise levels. As US becomes more widely used, physicians’ experience and skill levels will improve, further enhancing accuracy rates of US and achieving a win-win situation.

In the present study, tumor size and stage underestimation by US and MRI was associated with a larger pathological tumor size. Similarly, Xu et al. [8] and Vijayaraghavan et al. [31] found that MRI estimated tumor size more accurately for T1 stage tumors than for T2 and T3 stage tumors. This may be attributed to larger pathological sizes correlating with higher stages and a greater extent of tumor invasion into surrounding tissues. Traditional US and MRI may fail to accurately differentiate subtle infiltrative foci in the surrounding tissue, resulting in underestimation, which is a limitation that macroscopic examinations cannot easily address. It may be beneficial to combine some functional imaging techniques, such as diffusion-weighted MRI, to improve accuracy. However, in Mennella et al.’s study [20], the mean overestimation was higher in the T2–T3 stage tumors than in T1 stage tumors, which is in contradiction with our study results. This disparity may have resulted from their cases having different histopathological types (their study including more DCIS patients) and their including cases wherein MRI was performed after a biopsy procedure. Our findings suggested that clinicians should be cautious if the extent of physical examination exceeds the size reported by US or MRI, as this may indicate size underestimation by these methods. In surgical resection, it may be appropriate to expand the resection margins to avoid positive surgical margins.

Several studies indicated that histopathological type influenced the size concordance: invasive ductal carcinoma (IDC) with DCIS had higher discordance rate than pure IDC, and DCIS had higher discordance rate than invasive cancer [8, 9, 14, 20, 24]. Herein, unlike some previous studies, we could not identify the association between size discordance and histopathological type [16, 17]. Exclusion of DCIS may be the reason behind the negative association. Furthermore, discordance was suggested to be associated the molecular type of tumors [8, 15, 18]. In a study encompassing 6543 breast cancer patients, Stein et al. [15] showed that US was superior for HR-negative cancers. Similarly, our study showed that MRI stage overestimation was influenced by the PR status and that MRI provided more accurate results in PR-negative patients. Conversely, Yoo et al. [18] suggested that ER negativity was associated with MRI–pathology discordance. Furthermore, our study showed that molecular subtype was also not significantly associated with the tumor size or stage concordance rate. Based on previous studies and our study, we suggested that clinicians should be aware of the higher rate of discordance in tumor size or stage through US and MRI when preoperative pathology indicates IDC with DCIS, DCIS, or ER negative status. A comprehensive assessment of tumor size, including physical examination, is essential for precise personalized treatment.

This study had certain limitations. First, given its retrospective design, it was subject to inevitable selection bias. Second, the pathological size was determined by the initial pathological reports, and slides were not reviewed by pathologists in this study. As tumor size is a relatively objective criteria with minimal interpretive subjectivity, the impact of central review on tumor size measurement is minimal. Finally, measurements were determined by the radiologist and not computer-aided detection (CAD), which may have led to measurement bias.

## Conclusions

There were no significant differences in tumor size and stage estimation between US and MRI in early breast cancer patients, and US could be the first choice for tumor size and tumor staging. Tumor size and stage underestimation by US and MRI was associated with a larger pathological tumor size, and MRI-determined tumor stage was more accurate in PR-negative patients. Further research is needed to validate our findings with larger, more diverse patient populations across multiple-centers.

## Data Availability

The datasets used and/or analysed during the current study are available from the corresponding author on reasonable request.

## Abbreviations

US: Ultrasound
MRI: Magnetic resonance imaging
DCIS: Ductal carcinoma in situ
ILC: Invasive lobular carcinoma
SLN: Sentinel lymph node
ALND: Axillary lymph node dissection
DCE-MRI: Dynamic contrast-enhanced-MRI
MPR: Multiplanar reconstruction
NST: Invasive carcinoma of no special type
ER: Estrogen receptor
PR: Progesterone receptor
HER2: Human epidermal growth factor receptor 2
HE: Hematoxylin–eosin
SD: Standard deviation
TR: Repetition time
TE: Echo time
FOV: Field of view
T2WI: T2-weighted imaging
TNBC: Triple-negative breast cancer
IDC: Invasive ductal carcinoma
CAD: Computer-aided detection
